# Assessing the performance of a Loop Mediated Isothermal Amplification (LAMP) assay for the detection and subtyping of high-risk suptypes of Human Papilloma Virus (HPV) for Oropharyngeal Squamous Cell Carcinoma (OPSCC) without DNA purification

**DOI:** 10.1186/s12885-018-4087-1

**Published:** 2018-02-08

**Authors:** Mitchell G. Rohatensky, Devon M. Livingstone, Paul Mintchev, Heather K. Barnes, Steven C. Nakoneshny, Douglas J. Demetrick, Joseph C. Dort, Guido van Marle

**Affiliations:** 1 Undergraduate Medical Education, Faculty of Medicine & Dentistry, University of Alberta, Edmonton, AB Canada; 2 Section of Otolaryngology – Head and Neck Surgery, Department of Surgery, Cumming School of Medicine, University of Calgary, Calgary, AB Canada; 3 Department of Microbiology, Immunology and Infectious Diseases, Cumming School of Medicine, University of Calgary, Calgary, AB Canada; 4 Ohlson Research Initiative, Arnie Charbonneau Cancer Institute, Cumming School of Medicine, University of Calgary, Calgary, AB Canada; 5 Department of Pathology and Laboratory Medicine, Cumming School of Medicine, University of Calgary and Calgary Laboratory Services, Calgary, AB Canada; 6 Snyder Institute for Chronic Diseases, Cumming School of Medicine, University of Calgary, Calgary, AB Canada

**Keywords:** LAMP, HPV, OPSCC, Loop-mediated isothermal amplification, Oropharynx

## Abstract

**Background:**

Oropharyngeal Squamous Cell Carcinoma (OPSCC) is increasing in incidence despite a decline in traditional risk factors. Human Papilloma Virus (HPV), specifically subtypes 16, 18, 31 and 35, has been implicated as the high-risk etiologic agent. HPV positive cancers have a significantly better prognosis than HPV negative cancers of comparable stage, and may benefit from different treatment regimens. Currently, HPV related carcinogenesis is established indirectly through Immunohistochemistry (IHC) staining for p16, a tumour suppressor gene, or polymerase chain reaction (PCR) that directly tests for HPV DNA in biopsied tissue. Loop mediated isothermal amplification (LAMP) is more accurate than IHC, more rapid than PCR and is significantly less costly. In previous work we showed that a subtype specific HPV LAMP assay performed similar to PCR on purified DNA. In this study we examined the performance of this LAMP assay without DNA purification.

**Methods:**

We used LAMP assays using established primers for HPV 16 and 18, and new primers for HPV 31 and 35. LAMP reaction conditions were tested on serial dilutions of plasmid HPV DNA to confirm minimum viral copy number detection thresholds. LAMP was then performed directly on different human cell line samples without DNA purification.

**Results:**

Our LAMP assays could detect 10^5^, 10^3^, 10^4^, and 10^5^ copies of plasmid DNA for HPV 16, 18, 31, and 35, respectively. All primer sets were subtype specific, with no cross-amplification. Our LAMP assays also reliably amplified subtype specific HPV DNA from samples without requiring DNA isolation and purification.

**Conclusions:**

The high risk OPSCC HPV subtype specific LAMP primer sets demonstrated, excellent clinically relevant, minimum copy number detection thresholds with an easy readout system. Amplification directly from samples without purification illustrated the robust nature of the assay, and the primers used. This lends further support HPV type specific LAMP assays, and these specific primer sets and assays can be further developed to test for HPV in OPSCC in resource and lab limited settings, or even bedside testing.

## Background

Traditional risk factors for oropharyngeal squamous cell carcinoma (OPSCC) consist of alcohol and tobacco use. The epidemiology of OPSCC is changing, with human papillomavirus (HPV) associated OPSCC increasing in incidence despite a decline in use of alcohol and tobacco [1–5]. This change has important clinical implications, as HPV-positive patients have a better prognosis in terms of overall survival, progression-free survival, and local-regional recurrence when compared to HPV-negative patients [6–9]. Several investigators have suggested that intensive chemoradiation regimens currently used for HPV-positive OPSCC therapy may represent overtreatment resulting in unnecessary toxicity exposure and reduced quality of life outcomes [9–11]. Multiple randomized controlled clinical trials evaluating the efficacy of de-intensified treatment regimens for HPV-positive patients are currently on-going [12].

The prognostic and potential therapeutic value of determining the HPV status of OPSCC has led to the development of various methods to identify the presence or absence of high-risk HPV subtypes in the oropharynx. The two most commonly used methods of HPV detection are polymerase chain reaction (PCR) and p16 protein detection by immunohistochemistry (IHC) [13]. Amplification of target DNA by PCR is highly sensitive and specific, making it the current gold standard for detecting and subtyping HPV in the oropharynx [14, 15]. However, PCR is time consuming and costly, and the methods and infrastructure needed to perform PCR preclude an easy point-of-care assay. Immunohistochemical identification of p16 protein over-expression is clinically used as a surrogate marker of transcriptionally-active HPV in the oropharynx, because it is cost-effective and readily available [13, 16, 17]. The p16 protein is an endogenous tumour suppressor that is upregulated in response to HPV infection and subsequent oncogene expression. Given that p16 is variably expressed in uninfected tissues, the p16 IHC assay is less specific than PCR, which has the advantage of directly testing for the presence of HPV DNA [18–20]. Also, the p16 assay is still relatively expensive and has a slow processing time, because tissue slides must be prepared and reviewed by a pathologist. Combinations of various HPV assays have been studied, showing high sensitivity and specificity. Unfortunately, sample preparation requirements, increased costs, and excessive time make it unlikely that such combined approaches will be used clinically at the bedside or in resource limited settings [20–22]. Overall there is a need for a rapid, low-cost and accurate molecular diagnostic test for HPV detection and subtyping in the oropharynx.

Loop-mediated isothermal amplification (LAMP) is a cost-effective, robust and highly specific DNA amplification method that utilizes four to six primers and is driven by the *Geobacillus stearothermophilus* (*Bst*) polymerase [23, 24]. A positive LAMP reaction can be detected with the naked eye due to magnesium pyrophosphate precipitation, making gel electrophoresis and spectrophotometry unnecessary [25]. Extensive sample preparation and purification is also unnecessary, as the PCR inhibitors found in human tissues or samples do not affect *Bst* polymerase [26]. The LAMP assay has an additional benefit of being highly cost-effective, with estimates placing the running cost of a LAMP assay at less than 1/100th of a similar PCR assay [13, 26]. Importantly, LAMP has been successfully developed to detect various human pathogens including malaria and tuberculosis, in addition to HPV infection in cases of cervical carcinomas and external genital polypoid lesions [26–33]. In these studies, LAMP exhibits equivalent sensitivity and specificity when compared to PCR, while remaining lower cost and more rapid. There has been some work focused on the detection and typing of HPV for oropharyngeal carcinomas in subsites in the head and neck, [34]. Our previous work showed that a LAMP assay to detect and type HPV 16, 18, 31, and 35 in OPSCC, performed as well as a PCR on purified DNA [35]. However, we did not yet assess if the LAMP assay we used was able to detect HPV without DNA purification. The ability to skip the DNA isolation step would significantly simplify the diagnostic procedure. In this paper, we tested the LAMP assays without DNA purification. This work showed that the LAMP assay was robust and worked even in the presence of large amounts of impurities, which bodes well for developing this type of assay for use on clinical samples such as mouths swabs or tissue biopsies without time consuming DNA purification procedures.

## Methods

### HPV containing plasmids

Whole genome containing Plasmids were obtained from the Molecular Pathology Laboratory (Calgary Laboratory Services (CLS), Calgary, Alberta, Canada). pHPV-16 (ATCC 45113D), pHPV-18 (ATCC 45152D), pHPV-31 (ATCC 65446) and pHPV-35a/b (ATCC 40330/40331) plasmids were maintained in Top10 E. Coli (Invitrogen). DNA was purified using a QIAprep MiniPrep Kit (Qiagen). DNA concentrations were determined using a NanoDrop™ Spectrophotometer (Thermoscientific).

### Primer design for LAMP

The LAMP primers used in this paper were designed as follows. Previously established type-specific LAMP primers for HPV 16 and 18 were utilized [32]. Each set of primers consisted of two outer primers (F3, B3), two inner primers (FIP, BIP) connected by a TTTT linker, and two loop primers (LF, LB) to improve the amplification efficiency and detection threshold of the assay. Previously designed primers for HPV 31 and 35 did not contain two loop primers, thus new type specific primers were generated for these subtypes. FASTA sequences were obtained based on accession numbers for HPV 31 [GenBank: J04353.1] and HPV 35 [GenBank: M12732]. The E6/E7 promotor region of the HPV genome was selected as a target for primer design due to the conservation of the nucleotide sequence among individual HPV subtypes and relative variability of this nucleotide sequence between different subtypes. The E6/E7 promoter region is also vital to oncogenesis, therefore mutations in this region that would result in amplification failure would likely prevent carcinogenesis, thus making a HPV positive test more clinically significant. A nucleotide BLAST alignment was performed to ensure there was no cross-reactivity of the primers between subtypes. PrimerExplorer V4 (https://primerexplorer.jp/e/) software was utilized to design the primers for HPV 31 and 35. Table 1 shows the sequences of the various LAMP primers.

**Table 1:** Primers used for LAMP amplification of OPSCC specimens (5′ to 3′)

HPV 16^a^	F3	TCGGTTGTGCGTACAAAG
	B3	AGCCTCTACATAAAACCATCC
	FIP	TGGGGCACACAATTCCTAGT*CACACACGTAGACATTCGT
	BIP	TCAGAAACCATAATCTACCATGGC*ATTACATCCCGTACCCTCTT
	LF	CCCATTAACAGGTCTTCCAAAGT
	LB	CCTGCAGGTACCAATGGGG
HPV 18^a^	F3	AACGACGATTCCACAACA
	B3	CAACCGGAATTTCATTTTGG
	FIP	GTCTTTCCTGTCGTGCTCGGT*AGCTGGGCACTATAGAGG
	BIP	CGACGCAGAGAAACACAAGT*CTCTAAATGCAATACAATGTCTTG
	LF	CAGCACGAATGGCACTGG
	LB	TATTAAGTATGCATGGACCTAAGGC
HPV 31	F3	AGAAGAAAAACAAAGACATTTGG
	B3	CTCCTCATCTGAGCTGTC
	FIP	GTCTTCTCCAACATGCTATGCA*GAAACGATTCCACAACATAGG
	BIP	GAGAAACACCTACGTTGCAAGA*GGGTAATTGCTCATAACAGTG
	LF	ACGTCCTGTCCACCTTCCT
	LB	TGTGTTAGATTTGCAACCTGAGGCA
HPV 35	F3	TGCATGGAGAAATAACTACATTG
	B3	CGCCTCACATTTACAACAG
	FIP	TGTCACACAATTGCTCATAACAGTA*CAAGACTATGTTTTAGATTTGGAAC
	BIP	GCTCAGAGGAGGAGGAAGATAC*GACGTTACAATATTATAATTGGAGG
	LF	TATTGACGGTCCAGCTGGACAA
	LB	GGACAAGCAAAACCAGACA

* denotes a TTTT linker

^a^Saetiew et al. 2011

### LAMP reaction conditions

LAMP reagent concentrations were based on previously established reaction conditions. Total reaction volumes of 25 μL were utilized. LAMP reactions were performed using 8 units of *Bst* Polymerase 2.0 WarmStart (New England Biolabs), 1μL of template DNA (containing various copies of template plasmid), 1.4 mM of each dNTP, 0.5 M of Betaine, 6 mM of MgSO_4_ heptahydrate, 0.2μM of F3/B3 primers, 1.6μM of FIP/BIP, 0.8μM of LF/LB primer, 1X Amplification Buffer (New England Biolabs) and 10μL of ddH_2_O. Template DNA was exposed to a 95 °C boil step for 5 min prior to adding it to the reaction solution, which has been shown to increase the sensitivity of the LAMP assay [32]. The amplification reactions were carried out at 65 °C for 60 min, followed by 5 min at 80 °C to halt the reaction.

### Confirmation of positive LAMP reaction

A positive LAMP reaction was assessed using visual detection of change in turbidity, following corroboration with by standard TAE agarose (2%) gel electrophoresis at 100 V for 40 min and optical density (OD) measurement at 400 nm relative to negative control LAMP reactions.

### Determination of HPV copy number detection threshold

The concentration of positive control plasmids was determined using a NanoDrop™ Spectrophotometer (Thermoscientific) at 260 nm. Copy numbers of plasmid were determined using the calculated molecular weight of the various plasmid based on their base composition. Plasmids were diluted to a plasmid copy number of 10^6^ using nanopure nuclease free water. 10-fold serial dilutions in triplicates were then performed in to a final copy number of 10^0^ for each HPV subtype. LAMP reactions were then performed on each dilution.

### Determining the specificity of LAMP primers

The specificity of the type specific LAMP primers was tested using approximately 50 × 10^−12^g (i.e. 10^6^ copy numbers) of each plasmid. LAMP reactions were performed on each positive control plasmid using the various primers to ensure that there was no primer cross reactivity leading to false positive amplification, using this excess of target sequences.

### HPV amplification directly from cells using LAMP

HeLa 229 cells (ATTC#CCL2.1) were obtained from the Molecular Pathology Laboratory (Calgary Laboratory Services (CLS), Calgary, Alberta, Canada). HeLa cells are an immortalized human cervical carcinoma cell line containing multiple integrated fragmented copies (10 to 50 per cell) of HPV 18 DNA [36]. UM-SCC47 cells were obtained from the University of Calgary Arnie Charbonneau Cancer Institute cell bank (through Dr. Riabowol) and were donated by Dr. Thomas Carey, University of Michigan. UM-SCC47 is an oral SCC cell line containing multiple integrated copies (18 per cell) of HPV16 DNA [37]. A small quantity of frozen pelleted cells were obtained and utilized as template DNA for LAMP. Sample preparation consisted of a 5-min boiling step, with no further sample purification. Samples of HPV negative Hep3B cells (ATTC # HB-8064) (obtained by Calgary Laboratory Services (CLS), Calgary, Alberta, Canada) and Jurkat cells (Clone E6-1) (ATTC# TIB-152) were prepared in an identical fashion and utilized as HPV negative control.

To assess the performance of our LAMP assays in the presence of an abundance host cell DNA and impurities, thus mimicking a clinical sample, we used the HPV negative Jurkat cells lines. Jurkat cell pellets (2 × 10^6^ cells/100 μl (containing 0.25 μg/μl of DNA)) were mixed with the different HPV control plasmids and their amplification characteristics were compared to pure purified plasmid DNA. We also mixed Jurkat cell pellets with UM-SCC47 cell pellets to measure the effect of increasing competing cellular debris and DNA. Finally, purified DNA, obtained from an archived anonymized tissue block from a patient with HPV-16(+) OPSCC, was mixed with Jurkat cell pellets. The use of these samples was approved by the Health Research Ethics Board of Alberta – Cancer Committee (HREBA-CC) (approval reference number HREBA.CC-16-0181).Similar to the Hela and UM-SCC47 cells, these samples were boiled for 5 min without further DNA purification and used a template for different HPV LAMP assays.

## Results

### LAMP primers and HPV subtype specific amplification

Using PrimerExplorer V4 the previous published primers designed for HPV 16 and 18 [32] were validated for appropriate binding to their target sequences, and appropriate primers for HPV subtypes 31 and 35 were designed in a similar fashion. The primer sequence and target regions for each primer set are shown in Table 1. The primer design software was unable to generate a suitable LF primer from HPV 35 directly, therefore we manually designed a LF primer that we aligned directly with the LB primer to ensure there was no dimerization or cross annealing of the loop primers.

**Fig. 1:**
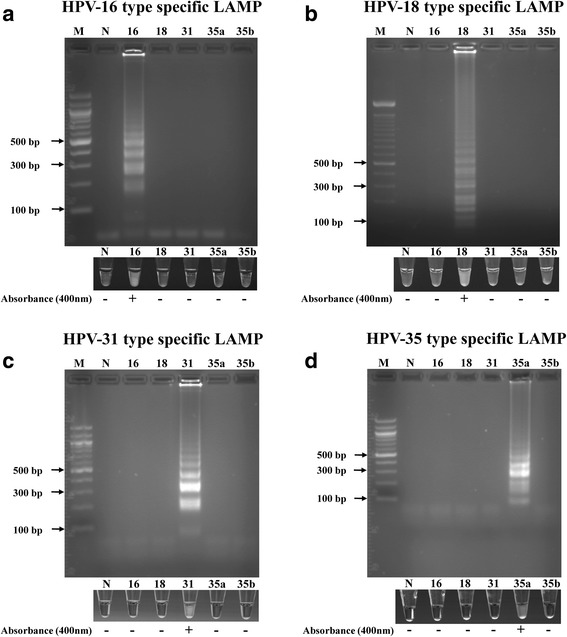
Specificity of the LAMP detection of different HPV types: Purified plasmid DNA containing genomic inserts extracted from HPV 16, HPV 18, HPV 31, HPV 35a, and HPV 35b were amplified using HPV 16 (**a**), HPV 18 (**b**), HPV 31 (**c**), and HPV 35 (**d**) type-specific LAMP primers. The detection of LAMP products was done by 2% agarose gel electrophoresis, visual assessment of precipitate formation in the reaction tube, and the presence (+) or absence (−) of spectrophotometer absorbance at 400 nm compared to negative controls. Lane M: 100 bp marker, lane N: negative control, and number: HPV types

To assess the HPV subtype specificity of the LAMP primers, we first tested high concentrations (10^6^ copy number) of each individual HPV subtype (16, 18, 31 or 35) containing plasmid, with the different primer sets. Figure 1 shows that each set of primers only amplified its specific subtype and no cross-amplification (amplification of other subtypes) was observed. Agarose gel electrophoresis, revealed the typical DNA ladder pattern of LAMP product in the positive samples. Positive samples showed distinct turbidity of the reaction mixture in the tube, as result of the magnesium pyrophosphate precipitation. This turbidity also resulted in increased OD 400 nm of the positive samples compared to the negative samples and controls.

### HPV copy number lower detection limit thresholds

**Fig. 2:**
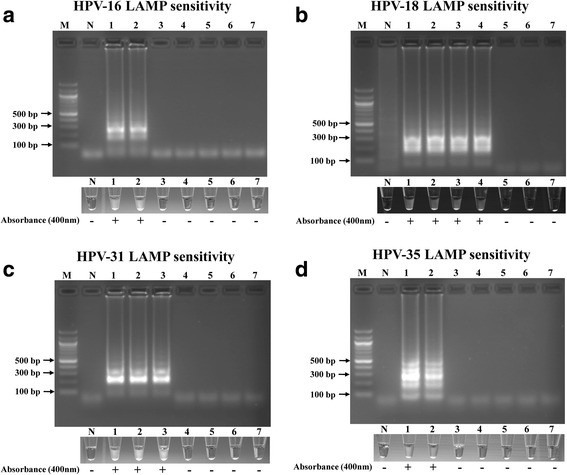
The sensitivity of the LAMP assay using HPV LAMP primers to detect HPV 16 (**a**), 18 (**b**), 31 (**c**), and 35a (**d**). LAMP reactions were assessed by 2% agarose gel electrophoresis, visual assessment of precipitate formation in the reaction tube, and the presence (+) or absence (−) of spectrophotometer absorbance at 400 nm compared to negative controls. Lane M: 100 bp marker, lane N: negative control, lanes 1-7: 10^6^ to 10^0^ copies of 10-fold serial dilutions of purified plasmid DNA containing genomic inserts extracted from HPV 16, HPV 18, HPV 31, HPV 35a

As the various HPV LAMP primer sets were subtype specific, we set forth to determine the lower detection limit for the various subtypes. Multiple 10-fold serial dilutions were made of the different HPV subtype genome containing plasmids (ranging from 10^6^ to 1 (10^0^) HPV genome copy numbers). Fig. 2 demonstrates the copy number detection threshold for each subtype. The LAMP assays easily detected 10^5^, 10^3^, 10^4^, and 10^5^ copies of HPV genome DNA for HPV 16, 18, 31, and 35, respectively, which is well within the clinical range of HPV genome copy numbers present in OPSSC tumors [38]. LAMP product positive reactions, as determined by agarose gel electrophoresis, displayed magnesium pyrophosphate precipitate turbidity that was easily discernible by eye (Fig. 2). This corresponded also to OD at 400 nm in the range of 0.102 to 0.407, compared to LAMP product negative and negative control samples (Fig. 2).

### LAMP HPV detection in human cells without DNA isolation

**Fig. 3:**
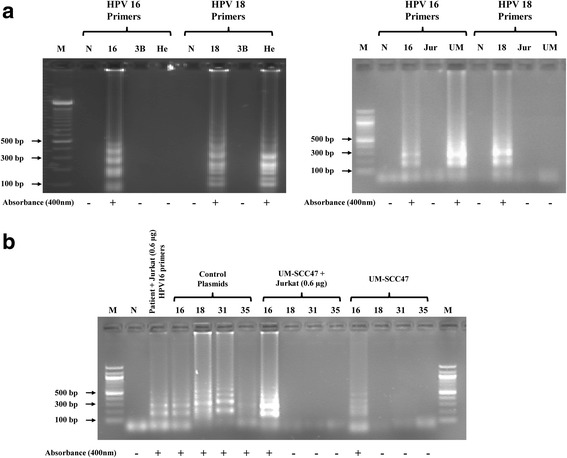
Direct LAMP detection of HPV in samples without DNA purification: **a** LAMP detection of HPV in boiled cells. Purified plasmid DNA containing genomic inserts from HPV 16 and HPV 18, as well as boiled Jurkat cells, HeLa cells, UM-SCC47 cells, and Hep3B cells, were amplified using HPV 16 and HPV 18 type-specific LAMP primers. HPV 16 and HPV 18 were detectable in the UM-SCC47 and HeLa cells respectively in type specific fashion, but not in the HPV negative Jurkat and Hep3B cells. The detection of the LAMP products was done by 2% agarose gel electrophoresis, assessment of precipitate formation in the reaction tube, and by presence (+) or absence (−) of spectrophotometer absorbance at 400 nm compared to negative controls. Lane M: 100 bp marker, lane N: negative control, lane 3B: Hep3B cells, lane He: HeLa cells, lane Jur: Jurkat cells, and number: HPV types. **b** LAMP detection of HPV in boiled UM-SCC47 cell samples in the presence and absence of excess Jurkat cells (DNA equivalents indicated), and in isolated HPV16 OPSCC patient tumor DNA with Jurkat cells. In the presence of Jurkat cells HPV DNA from boiled UM-SCC47 cells and from isolated HPV16 OPSCC patient tumor DNA could be easily amplified in a type specific fashion. These results indicate that LAMP reactions perform robustly in samples without the need for any DNA purification, similar to what would be the case for a tumor swab or biopsy. The detection of the LAMP products was done by 2% agarose gel electrophoresis, assessment of precipitate formation in the reaction tube, and by presence (+) or absence (−) of spectrophotometer absorbance at 400 nm compared to negative controls. Lane M: 100 bp marker, lane N: negative control, and number: HPV types

The LAMP assays were able to detect levels of HPV DNA relevant to the amounts found in clinical samples. We continued testing to determine if our primers were able to detect HPV DNA in tissue culture cells without DNA purification. Figure 3 demonstrates successful, subtype specific amplification of HPV 18 directly from HeLa cells and of HPV 16 from UM-SCC47 cells without sample DNA purification, similar to our observations with purified HPV plasmid. To further simulate a clinical sample such as a mouth swab or tissue biopsy, we mixed Jurkat cell pellets with purified HPV plasmids, as well as purified DNA obtained from an archived anonymized HPV 16 positive OPSSC sample used in another study by our group [35]. In addition, we mixed UM-SCC47 cells with excess of Jurkat cells, to mimic extra competing DNA and excess of potential tissue inhibitors of the LAMP reaction. In all cases. In all cases, our LAMP assays successfully amplified HPV in a subtype specific manor (Fig. 3b). These results indicate our HPV type specific LAMP assays are robust, and the assays are not inhibited by impurities present in unpurified DNA samples.

## Discussion

LAMP for the detection of HPV in cervical carcinoma has been studied previously, with LAMP kits now available for commercial use [33]. HPV related OPSCC is an emerging disease entity with the potential for application of LAMP to detect high-risk subtypes of HPV for OPSCC. In our previous work, we tested primers and LAMP assays for HPV16,18,31 and 35 on purified DNA from archived biopsy samples from patients with OPSCC [38], and that study showed that our LAMP assay performed similar to established PCR protocols, for detecting and typing HPV in OPSCC. However, in order to assess if they would have potential for use in LAMP tests without DNA purification, we needed to examine the performance of our LAMP assays and primers in more detail under different conditions.

All primer sets. For HPV16, 18, 31, and 35 successfully amplify from target plasmid DNA. The reaction conditions choosen were optimized to prevent nonspecific amplification, and included titration of MgSO4 and reaction temperature parameters. It is noteworthy that false positives due to non-specific amplification were initially seen when the primers were reconstituted using water instead of TE Buffer (Data not shown). It is possible that over time, reconstitution in water resulted in primer depurination and degradation due to the relative acidity of our deionized water supply. Nonspecific amplification was prevented when fresh primers and TE buffer were utilized.

The reaction was shown to be able to detect viral DNA down to a copy number of 10^5^ for HPV 16, 10^3^ for HPV 18, 10^4^ for HPV 31, and 10^5^ for HPV 35 with 100% specificity. These results are consistent with previously established sensitivities of LAMP [32]. There are differences in the lower detection limit of the different subtypes. We believe that this is due to the different priming efficiencies of the different primer sequences. One of the goals of this work was to design and test LAMP primers that are type specific. The priming specificity may go at the expense of priming efficiency, which may in turn affect the lower detection limit. However, viral copy number varies in vivo in relation to cell type, HPV subtype and grade of HPV related dysplasia. HPV positive cervical cytobrush swabs have been shown to contain quantities of DNA well above these detection thresholds, with for instance an approximate median HPV-16 copy number of 6 × 10^5^ copies, among low-grade dysplasia samples [39]. In head and neck cancers, in particular in tonsillar tissues, similar copy numbers have been found [38]. Therefore the detection thresholds demonstrated in this study would be more than sufficient and shows the potential of LAMP for the detection and typing of HPV, even with the quantities of HPV infected tissue, such as that obtained by an oropharyngeal swab.

The E6-E7 proteins are important for oncogenesis and reverse transcriptase PCR (RT-PCR) protocols detecting the expression E6-E7 encoding mRNAs in a tumor are considered a good indicator of HPV induced/associated oncogenesis. RT-LAMP procedures to detect RNA are available [40, 41]. However, detecting RNAs directly from tissues may be problematic due to degradation by the RNases present in the sample, and may require nucleic acid purification or some extra sample treatment step. Our LAMP primer target the E6/E7 promoter region, which allowed for the design of HPV type specific LAMP primers. More importantly, we also chose that region as we believe that it also increase the possibility that the HPV DNA detected by our LAMP assays still has oncogenic potential as they would still be able to express the HPV *e6-e7* gene products. This would offer a similar benefit as testing for E6-E7 encoding mRNA but without the need for further sample preparation, which overall simplifies the assay. Of course these different assays will need to be tested side by side, which is part of our follow up work.

Confirmation of a positive LAMP reaction was consistent among visual detection, optical density measurement and gel electrophoresis. Visual detection is the most simple and cost-effective method of confirmation, and our results confirmed that visual assessment of relative turbidity alone is reliable and consistent. This further demonstrates the potential of LAMP as a bedside clinical diagnostic test; because LAMP reaction tubes can remain closed following amplification, thereby preventing the risk of contamination of one’s clinical workspace with large amounts of HPV DNA amplicons. Concerns over contamination can also be addressed by utilizing dUTPs rather than dNTPs with the LAMP reaction. *Bst* polymerase can use dUTP when amplifying a target gene, so that all contaminating amplicons can be degraded with the addition of uracil N-glycosylase (UNG) prior to LAMP amplification. Recent studies have shown that the addition of UNG during sample preparation while using dUTP during LAMP amplification is an effective method for preventing contamination [42]. The use of lyophilized UNG could also be explored, along with the use of the assay without refrigeration, due to the long half-life of *Bst* polymerase enzyme at ambient temperature.

The main goal of our study, was to test our LAMP assay and primers (for HPV16, 18,31 and 35) and compare and analyze how they would perform without any DNA purification. In our previous work, we have tested these primers on purified DNA from archived biopsy samples from patients with OPSCC [35], and this study showed that our LAMP assay performed similar to established PCR protocols, for detecting and typing HPV in OPSCC. In this study, we showed we could use our LAMP for detection and typing of HPV directly on different types of cells mimicking clinical samples, without any intermediate DNA purification steps. Even with adding excess of cells compared to target DNA derived from both HPV containing plasmid DNA constructs as well as patient derived DNA, we were able to detect HPV in a type specific fashion with similar lower detection limits as purified DNA. The latter shows clearly that even with amounts of “contaminants probably beyond what is found in a biopsy of tissue swap, our type specific LAMP assays are robust. In our previous paper [35] using purified DNA, we were able to compare a PCR approach to a LAMP approach. We did not test PCR vs LAMP in our current study, as in our experience the PCR approach we use on purified DNA in our previous work does not appear to work on unpurified DNA (data not shown). This may need to be confirmed further but those observations would suggest our LAMP approach is probably better suited for unpurified samples than our PCR approach. Known inhibitors of Taq polymerase, the enzyme that drives standard PCR reactions, include heme, collagen, melanin and IgG [26, 29]. *Bst* polymerase is unaffected by these and other inhibitors that prevent PCR mediated DNA amplification. LAMP has been performed reliably on CSF, serum and heat-treated blood with no further sample preparation [43]. Not having to purify DNA from samples, means that LAMP requires minimal sample preparation but remains reliable with a sensitivity and specificity comparable to standard PCR methods. HPV31 and 35 positive patient samples are relatively rare at our clinic, thus we had to limit our work for these subtypes to cell lines and samples mimicking clinical sample. The next step will be direct testing or LAMP conditions and primers on larger cohorts of patients, using bedside or operative samples collected via cytobrushes or swabs. Direct and rapid testing of samples will expedite the prognostic and diagnostic pipeline, which could positively affect clinical outcomes. Although, we did not investigate time to positive test results directly and systemically, on average we take 1 h for DNA purification plus 3 h for PCR setup and running the PCR, compared to 1 h to 1.5 h to perform a LAMP reaction including setup. This would suggest considerable time savings, even if one includes 30 min to examine and confirm presence of LAMP products using agarose gel electrophoresis. Careful examination of all these factors such as time to positive test, detection limits, and the applicability of LAMP testing in clinical and resource-limited settings warrants further investigation in the context of OPSSC, as we believe LAMP has considerable diagnostic potential for HPV testing in OPSSC.

## Conclusion

This study demonstrates the potentialof LAMP test for subtyping of HPV infection in OPSCC without DNA purification. This technology could be used in a clinical setting to identify oropharyngeal lesions infected by high-risk subtypes of HPV DNA. The primers we designed for different HPV subtypes reliably amplified target DNA. This study demonstrated that LAMP has the specificity and copy number lower detection limits of PCR without the requirement for extensive sample preparation and expensive detection methods. The assay is quicker than PCR and would require minimal infrastructure. LAMP should be further developed for use at the bedside and in resource poor settings for the rapid diagnosis and subtyping of HPV in OPSCC.

## Abbreviations

Bst: *Geobacillus stearothermophilus*
HPV: Human papillomavirus
IHC: Immunohistochemistry
LAMP: Loop mediated isothermal amplification
OPSCC: Oropharyngeal squamous cell carcinoma
PCR: Polymerase chain reaction
UNG: uracil N-glycosylase
